# Epigenetic regulation of the honey bee transcriptome: unravelling the nature of methylated genes

**DOI:** 10.1186/1471-2164-10-472

**Published:** 2009-10-14

**Authors:** Sylvain Foret, Robert Kucharski, Yvonne Pittelkow, Gabrielle A Lockett, Ryszard Maleszka

**Affiliations:** 1 Centre for Bioinformation, Mathematical Sciences Institute, The Australian National University, Canberra ACT 0200, Australia; 2 Research School of Biology, The Australian National University, Canberra ACT 0200, Australia

## Abstract

**Background:**

Epigenetic modification of DNA via methylation is one of the key inventions in eukaryotic evolution. It provides a source for the switching of gene activities, the maintenance of stable phenotypes and the integration of environmental and genomic signals. Although this process is widespread among eukaryotes, both the patterns of methylation and their relevant biological roles not only vary noticeably in different lineages, but often are poorly understood. In addition, the evolutionary origins of DNA methylation in multicellular organisms remain enigmatic. Here we used a new 'epigenetic' model, the social honey bee *Apis mellifera*, to gain insights into the significance of methylated genes.

**Results:**

We combined microarray profiling of several tissues with genome-scale bioinformatics and bisulfite sequencing of selected genes to study the honey bee methylome. We find that around 35% of the annotated honey bee genes are expected to be methylated at the CpG dinucleotides by a highly conserved DNA methylation system. We show that one unifying feature of the methylated genes in this species is their broad pattern of expression and the associated 'housekeeping' roles. In contrast, genes involved in more stringently regulated spatial or temporal functions are predicted to be un-methylated.

**Conclusion:**

Our data suggest that honey bees use CpG methylation of intragenic regions as an epigenetic mechanism to control the levels of activity of the genes that are broadly expressed and might be needed for conserved core biological processes in virtually every type of cell. We discuss the implications of our findings for genome-scale regulatory network structures and the evolution of the role(s) of DNA methylation in eukaryotes. Our findings are particularly important in the context of the emerging evidence that environmental factors can influence the epigenetic settings of some genes and lead to serious metabolic and behavioural disorders.

## Background

In eukaryotes, gene activity is regulated by several interacting systems operating at a number of levels, including epigenetic modifications of DNA [1,2]. One such mechanism is DNA methylation that has the capacity to establish and maintain diverse patterns of gene expression from the same genome under specific temporal, spatial and environmental conditions [3]. This ability to selectively modulate gene activity is a key evolutionary invention that is critical to generating the variety of cell types and phenotypic polymorphism in eukaryotic species. DNA methylation is widespread among eukaryotic species, but both the level and overall pattern of methylation vary noticeably in different lineages [3,4]. It is believed that this post-replication modification of genomic DNA provides a link between genomes and environment and may result in a phenotypic change that is heritable, but does not involve DNA mutation [5,6]. In mammals, DNA methylation has been implicated in tissue-specific gene regulation, parental imprinting and silencing of transposable elements [3,5,7]. A recent integrated study of human genome-wide tissue-specific DNA methylation profiles confirmed the negative correlation between gene expression and methylation at CpG-containing promoters [8]. In contrast, gene-body methylation has been found to be positively correlated with gene expression. A strong relationship between intragenic methylation and transcription has also been uncovered in *Arabidopsis *[9].

Until recently, genomic methylation in invertebrates has received less attention [10-12] and its biological role was considered somewhat controversial [12-14]. One impeding factor in these earlier studies was the lack of technological sophistication that would allow evaluating the methylomes in species with very low and variable methylation levels. In recent years, the rapid progress in genomic sequencing revealed that 'vertebrate-like' enzymatic machinery required for CpG methylation is encoded by many invertebrate genomes, including several insect genomes [15-18]. More importantly, recent experimental data in honey bees show that this system is fully functional [16] and is utilized to generate nutritionally-controlled phenotypic polymorphism that lies at the core of social organization of this species [19]. In addition, broad expression patterns of DNA methyl-transferases (Dnmts) in honey bees that include embryos and the adult nervous system [19], suggest that epigenetic controls of genome activities also play important roles in early development and in brain plasticity. Recent studies on DNA methylation in another invertebrate, *Ciona intestinalis*, provided compelling evidence for the existence of distinct methylated domains across the genome that co-localize with around 60% of transcription units encoding evolutionarily conserved, infrequently transcribed genes [20]. These authors proposed that CpG methylation functions as a mechanism suppressing spurious transcriptional initiation of rarely transcribed genes. These findings raise a number of important questions. Do all invertebrates share a similar pattern of genome methylation? Does the invertebrate mode of genome methylation represent a primordial function of DNA methylation in animals? Are the methylated genes in honey bees important for social behaviour and if so, are they a special subset of the genome? Are there any commonalities in their predicted biological functions and/or structural characteristics?

As part of our effort to understand the biological significance of genome methylation in honey bees we combined bioinformatic analyses, microarray-based transcriptional profiling and bisulfite sequencing to determine if methylated genes in honey bees can be identified and organised in functional categories that would shed more light on their biological importance, in particular in the context of the evolution of eusociality, and the role(s) of DNA methylation in animals. We find that broadly expressed genes, typically classified as 'maintenance genes', fall into the methylated category, whereas distinctly regulated genes are not predicted to be methylated. Our data demonstrate that in *Apis*, gene activities required for core biological processes are controlled, at least partly, by epigenetic means. We discuss the implications of our findings for the origins of DNA methylation patterns in animals and their contribution to complex regulatory networks.

## Results

### Predicting the methylation status of transcription units in Apis mellifera

**Figure 1:**
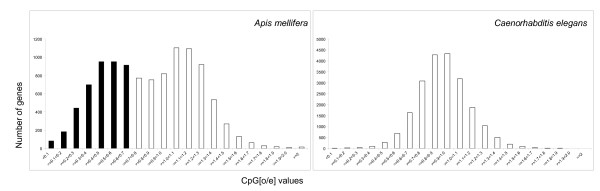
**Histogram showing the frequency of all annotated Apis genes with CpG [o/e] frequencies between 0 and 2**. For comparison, a similar analysis was performed on the nematode (*C. elegans*) genome that is not methylated due to the lack of the genes encoding DNA methyl-transferases. The honey bee CpG-deficient genes (CpG [o/e] <0.7) expected to be methylated are labelled in black. We used the honey bee official set of 10,742 genes available at BeeBase . The *y*-axis depicts the number of genes with the specific CpG [o/e] values given on the *x*-axis.

Methylated cytosines are frequently deaminated to uracil that is subsequently converted to thymidine after DNA repair. As a result of this process methylated CpGs are expected to decrease in abundance over evolutionary time, and the ratio of observed to expected CpGs can be used to predict methylated and unmethylated genomic regions [20,21]. Figure 1 shows the frequency of all annotated protein coding genes in *Apis *with CpG [o/e] frequencies between 0 and 2. For comparison, the contrasting distribution of all protein coding genes in a nematode lacking the DNA methylation system is shown in panel B. The bimodal distribution in *Apis *is indicative of two distinct groups: one representing CpG deficient genes with mean CpG [o/e] of around 0.55, and the second containing genes with the CpG [o/e] frequency mean ratio of 1.15. We estimate that around 35-40% of the 10,742 annotated *Apis *genes belong to the CpG deficient group and are expected to be methylated at CpGs located within their coding regions. To confirm these predictions we selected 14 genes for detailed analyses by bisulfite sequencing. As shown in table 1, in all cases the genes with CpG [o/e] ratios <0.8 have been confirmed to be methylated. In contrast, no CpG methylation has been detected in the selected exons of genes with CpG [o/e] > 1.0 suggesting that these genes are either not methylated or their methylation is restricted to a precisely defined developmental stage or a specialised group of cells. Interestingly, two out of six analysed genes with CpG [o/e] > 1.0 have classic CpG islands in the upstream region from the ATG start codon (table 1).

**Table 1:** Selected genes with contrasting CpG [o/e] ratios analysed by bisulfite sequencing

**Genes ubiquitously expressed predicted to be methylated**						
**Gene ID or** **Common name**	**OGS2 ID or** **GenBank ID**	Predicted function and expression patterns*	**CpG [o/e]**	**CpG island**	**Exon(s) analyzed**	**Predicted Methylation status**

DCTN4 dynactin p62	XP_001121083	A subunit of the Dynactin complex	0.62		4-6	Confirmed

MTM myotubularin myopathy related protein 9	GB19180	Multiple cellular functions, in humans, brain protein linked to neuropathies	0.57		5 7	Confirmed

Histone methyltransferase	GB13959	Histone modifications	0.58		4	Confirmed

Nadrin	GB16176	A novel GTPase-activating protein	0.47		10	Confirmed

PKCbp1 Receptor of activated prot.kinase C	GB12499	The ligand, PKC, is involved in learning, such as spatial learning in rats	0.65		5-6	Confirmed

TBP TATA-box binding protein	GB19036	A general transcription factor for RNA polymerase I, II and III.	0.35		1-2	Confirmed

Casein kinase II beta	GB12504	Involved in circadian rhythm, brain development	0.42		3	Confirmed

Swiss cheese\NTE neuropathy target esterase	GB10208	Involved in apoptosis and brain development	0.68		9	Confirmed

**Genes with restricted patterns of expression predicted to be unmethylated**						

GLOX glucose oxidase	GB19418	FAD flavoprotein oxidoreductase Restricted pattern of expression (very high in HP gland)	1.14	YES	Promoter 7-8	Confirmed

VHDL lipid transporter	GB15055	Larval-specific, very high density lipoprotein	1.39		13-15	Confirmed

OBP13 odorant binding protein	GB18363	Expressed during late larval stages and in pupae [49]	1.17		2-5	Confirmed

Not available	RIKEN EST DB777978	Unknown function, highly expressed in worker head	1.96			Confirmed

ImpL3-like L-lactate dehydrogenase	GB13882	Larval gene upregulated in worker larvae	1.33	YES	3-4	Confirmed

Squid RNP-CS RNA-binding domain protein	GB15796	Required for the correct localization and translational regulation of the *gurken *message	1.22		5-7	Confirmed

*Expression patterns are based on published data and our microarray analyses. Ubiquitous: expressed under all examined conditions. Restricted: typically expressed in one or two tissues. CpG islands were predicted using cpgplot at

### Genome-wide profiling of gene expression in Apis using oligo microarrays

To ask whether the methylation status of a gene could be correlated with its expression pattern, we used the honey bee genomic array to visualise the transcriptional activities in six functionally diverse tissues: brains, antennae, ovaries, thoraces, mixed larvae and hypo- pharyngeal (HP glands). Blank arrays were included for validation purposes. The design of long oligos for this new microarry platform is largely based on computer-generated gene models that yield around 70% accuracy [22,23]. In order to evaluate the biological power of this tool we included a pool of RNAs representing virtually all tissues and developmental stages (RNA cocktail) to determine the extent to which the oligos selected for the array correspond to transcribed sequences that can be visualized with this technology. Additional file 1 shows the distribution of the proportion of the 'detectable' spots for each array and each channel under different experimental conditions. In this section we call 'present', the spots having an intensity value greater than the 95^th ^percentile of the null distribution derived from the negative controls. On the blank arrays, this proportion varied between 1.6% and 11.2% indicating that this method has a low rate of false discovery. The observed variability in the proportion of the present spots (additional file 1), even between the two channels of a single array is often associated with two-colour microarray platforms [24]. In accord with other studies (see for example ref [25]) we found that appropriate RNA pooling significantly improves the reproducibility between the experiments. The most consistent data were obtained from the antennal RNA sample that represents a pool of RNAs extracted from 100 antennae or 50 individuals (additional file 1).

### Tissue-specific and ubiquitous profiles of expression

In this section, we call a gene 'expressed' if its cDNA probes have a median expression probability greater than 95% (see methods). The percentage of genes expressed under various experimental conditions are summarised in table 2. As in the previous section, the low number of oligos hybridizing on the blank arrays (0.84%) confirms that our method results in a low rate of false positives. Three out of four positive controls were found in all experiments, but one was not detected in each of the three experiments: HP gland, larvae and thorax. This result suggests that our approach tends to slightly underestimate the number of expressed genes, but with only four positive controls, it is difficult to conclude with certainty to what extent false negatives are produced. As expected, complex tissues (brains, antennae and ovaries), show the highest level of transcriptional activity by expressing 60-70% genes, whereas a highly specialised organ (HP gland) expresses only 14% of genes. Thoraces and larvae show an intermediate level of gene activity (40%). Almost 70% of oligos hybridized to the RNA cocktail. To assess the difference in CpG [o/e] frequencies between ubiquitous and condition-specific transcripts, we first compared these two categories and observed that the proportion of transcripts with CpG [o/e] frequencies smaller than 1.0 was significantly larger in the category of ubiquitously expressed genes (p = 7.9e-111, Fisher exact test). We then contrasted the ubiquitous and condition-specific transcripts with the entire collection of transcripts. The genes with CpG [o/e] frequencies smaller than 1.0 were found to be over-represented in the ubiquitous category (p = 3.7e-77, hypergeometric test), whereas the genes with a CpG bias larger than 1.0 were over-represented in the condition specific category (p = 3.0e-51).

**Table 2:** Percentages of cDNAs, positive controls and negative controls expressed under various experimental conditions (see materials and methods for more details).

**Condition**	**cDNA (%)**	**Positive controls (%)**	**Negative controls (%)**
Antennae	70.45	100	0
Brains	59.82	100	0
HP Gland	14.47	75	0
Thoraces	38.18	75	0
Ovaries	71.66	100	0
Larvae	49.22	75	0
RNA Cocktail	67.04	100	0
Blank	0.84	0	0

From these combined data we conclude that 11,684 probes (86.94%) are expressed in at least one experimental condition. The remaining non-hybridizing oligos have been either assigned to non-transcribed genomic sequences, or their hybridization intensities fell below the acceptable confidence level.

### Condition-specific and ubiquitous genes

**Figure 2:**
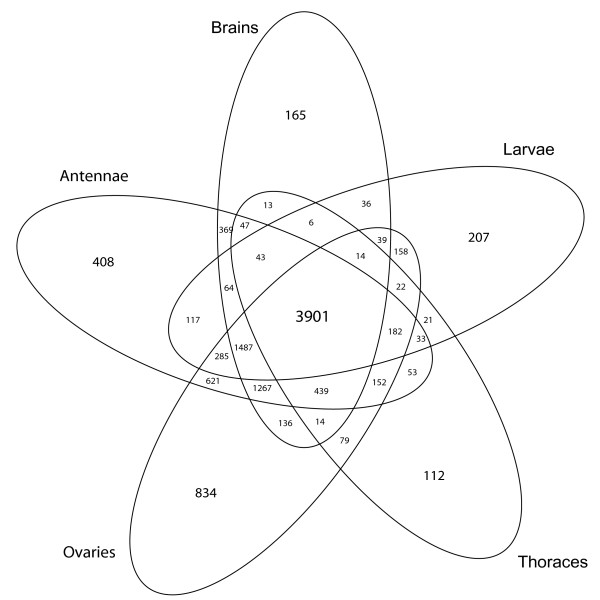
**Venn diagram showing the overlap of gene expression profiles between five experimental conditions: antennae, brains, thoraces, ovaries and larvae**. Only five conditions were selected for this diagram (it is impossible to plot a Venn diagram in two dimensions with more than five sets using ellipses [48]). ArrayExpress accession: E-MEXP-2093.

**Figure 3:**
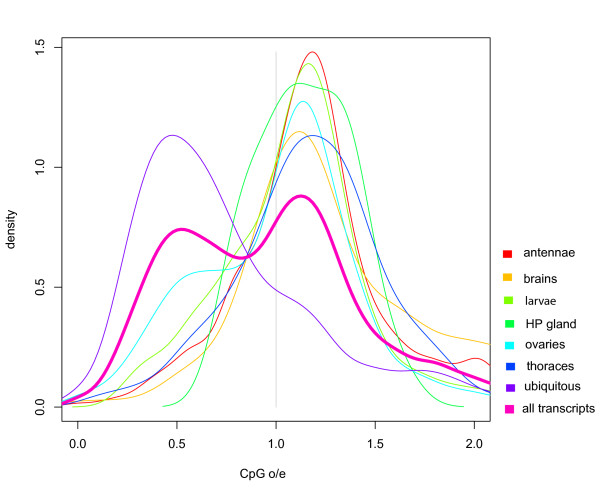
**Distribution of CpG bias in: i) all Apis predicted transcription units, ii) the ubiquitously expressed genes, and in iii) the condition-specific genes (HP Gland - hypopharyngeal gland)**.

Figures 2 shows the number of expressed genes identified in various tissues and the number of shared transcripts between our experimental conditions. The overlap generated by this analysis represents our ubiquitous set of genes. To further illustrate the relationship between methylated and unmethylated genes we generated CpG frequency plots for each of the three classes: i) condition specific genes, ii) the ubiquitous set of genes, and iii) all *Apis *predicted transcripts. The results are shown in figure 3. A characteristic bimodal shape of the CpG bias distribution for all predicted transcripts as already shown in figure 1 is also illustrated against the other profiles. The first peak corresponds to genes depleted in CpG dinucleotides, whereas the second peak comprises genes with a mean CpG bias value slightly larger than one. The distribution of ubiquitous genes largely overlaps with the first peak of all predicted transcripts and comprises mostly genes with low CpG dinucleotide content. In contrast, the distribution of condition specific genes closely matches the second peak representing high CpG ratios. The same trends were observed when different presence/absence thresholds, at which genes are considered expressed, were used (Additional file 2). The number of ubiquitous genes (~3900) revealed by microarraying (figure 2) is almost identical to the number of methylated genes (~4000) identified by the CpG plot shown in figure 1. Together with the detailed analysis of selected genes presented in table 1, these results demonstrate that methylated transcription units in *Apis *are broadly expressed and are likely to be active in all tissues.

### What are the functions provided by genes identified in this study as ubiquitously expressed and putatively methylated?

**Figure 4:**
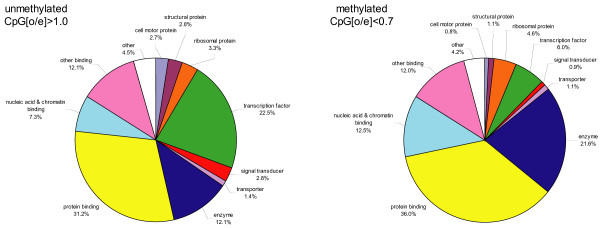
**Functional categorization of methylated and unmethylated genes based on Gene Ontology (GO) classification**. GO terms were assigned to honey bee predicted proteins using the corresponding GO terms of their BLASTP hits in the RefSeq *Drosophila *protein database. If the best hit did not have any associated GO terms, the best subsequent hit with associated GO terms was used and no GO terms were assigned to honey bee proteins that did not have any GO annotated hit with an e-value smaller than 1e-5. For illustration purposes only molecular function level 3 ontology terms (where Level 0 = root = Gene_ontology) were selected and grouped into larger categories. See additional file 4 for more details.

We used the Gene Ontology (GO) classification to sort out the methylated and un-methylated genes into broad functional categories and found significant differences in a number of categories (figure 4). As expected, genes encoding essential metabolic and energy transfer enzymes are more abundant in the methylated group than in the unmethylated group (21.6% versus 12%, p = 7.15*10^-13^, hypergeometric test). One example of a methylated highly conserved gene is triose-phosphate isomerase (TPI, EC 5.3.1.1) that plays a key role in glycolysis and is essential for efficient energy production. TPI not only provides a vital cellular function, but also is found in virtually all living creatures. Only non-glycolytic bacteria, like ureaplasmas, lack TPI. Other functional categories over-represented in the methylated set are nucleic acid and chromatin binding (12.5% v. 7.3%, p = 9.31*10^-7^). These highly conserved proteins may regulate the translation of RNA, and post-transcriptional events, such as RNA splicing and editing, nucleolytic cleavages and chromosome packaging among other functions. In contrast, the methylated group has significantly fewer genes encoding transcription factors than the unmethylated group (6% v 22.5%, p = 2.64*10^-40^). Furthermore, the smaller fraction of methylated TFs appears to be of a universal type belonging to the general transcription factor (GTF) category, as exemplified by the TATA-binding protein (TBP) that is used by all three RNA polymerases. Likewise, genes associated with signal transducing activities are under-represented in the methylated category (0.9% versus 2.8%, p = 4.60*10^-5^). Thus, in spite of an unrefined meaning of GO classification, the general functional categories revealed by this approach are surprisingly relevant and strongly suggest that methylated genes in honey bees encode conserved proteins involved in core cellular processes. We note that the honey bee GO diagrams are very similar to those generated by an analogous analysis of 16,310 *Arabidopsis *genes, 26% of which are predicted to be methylated [9].

## Discussion

One unifying theme of the honey bee methylome is the broad pattern of expression of methylated genes indicating that gene activities required for the core cellular functions might be controlled, at least partly, by epigenetic means. Although these ubiquitously expressed genes may not represent the nominal size of the 'housekeeping' transcriptome in this organism, it seems likely that they are constitutively expressed in time and space. Such permanently activated genes providing 'maintenance' functions required by virtually all cells have been typically described in the past as unregulated. However, it has been suggested that in spite of their permanent activation the 'housekeeping' genes might not be required at the same level throughout development [26], or under changing environmental conditions. Indeed, evidence suggests that even most stable transcripts are sensitive to both biotic and abiotic external influences [27,28]. Our data add more weight to the notion that the activities of 'housekeeping' transcripts and their products might be modulated by epigenetic means. Such a mechanism may also exist in other organisms [9,20] suggesting that a direct relationship between gene methylation and transcription is a widely spread phenomenon in both the animal and plant kingdoms.

In mammals, the majority of promoters driving the 'housekeeping' genes are associated with CpG islands [29]. These genomic regions containing a high frequency of CG nucleotides are typically not methylated with the exception of CpG islands on the inactive X chromosome and in disease situations. In contrast to mammals, the broadly expressed genes in *Apis *do not have CpG islands, whereas two out of six unmethylated genes with restricted patterns of expression selected for our detailed analyses (GOX and Impl3) are associated with classic CpG islands (table 1). GOX is stringently regulated and its expression is exceptionally high in the HP gland of nurse bees, whereas Impl3 is predominantly a larval gene, and its differential expression in worker and queen larvae is part of a network that determines the reproductive fate of female bees [19]. Although Impl3 is not directly methylated (table 1), its expression is reduced in Dnmt3-silenced larvae or by feeding royal jelly [19,30], suggesting that both unmethylated and methylated genes might be influenced by epigenetic controls in highly interconnected regulatory network structures. In honey bees, diet-induced changes in methylation levels lead to metabolic acceleration and increased growth driven by global, but relatively subtle changes in the expressional levels of a large number of genes [19,30]. These initial changes are later followed by the activation of more specific pathways to lay down caste-specific structures, such as pollen collecting combs on workers' legs that are built during pupal stages. Thus, instead of inventing two separate developmental blueprints, the bees differentially use one common plan to produce two distinct organismal outputs [17]. Here the entire network rather than its individual components evolved to create an alternative developmental trajectory. This might occur if a given phenotype is biologically regulated by large numbers of subtle gene expression differences that act additively, in cascade leading to a major change in the topology of a global network of interacting genes ([31-34] and references therein). A recent *in silico *analysis confirms that queen-worker transcriptional differences are associated with genes showing distinct CpGo/e ratios [35]. The epigenetic regulation of phenotypic polymorphism in honey bees is an example of the adaptive value of phenotypic plasticity that was the driving force in generating the reproductive division of labour in social insects.

Like in other invertebrates [10,20] the global level of genome methylation in *Apis *is low and appears to be restricted to CpGs residing in coding exons [16,36]. It has been argued [14,10] that global methylation, a hallmark of vertebrate genomes, arose within the phylum Chordata at the time when vertebrates originated, and was a major source of innovation at the genomic level. However, Regev et al [11] concluded that methylation, originally used as a general repressor of genomic parasites, was recruited to perform gene regulatory functions well before the transition from invertebrates to vertebrates. One possibility is that transcriptional regulation by DNA methylation is an ancient mechanism of gene control that was adequate for primordial metazoan species with limited cell type and tissue repertoires. As animal evolution progressed, novel regulatory mechanisms operating via promoters and sequence-specific transcription factors (TFs) were invented to generate both the developmental sophistication and cellular diversity that characterise modern animals. As a result, organismal complexity is largely instantiated at the level of differential gene expression that evolved by combining the specific TFs, differential splicing, non-coding RNAs, chromatin remodeling and epigenetic modification of genomic DNA by methylation [1]. In this context, the lack of an obvious correlation between gene number and apparent morphological and behavioral complexities of diverse organisms in different phyla [37] is not surprising. While the combinatorial interactions of TFs and their targets are now well understood [38,39], the role(s) of epigenetic modifications in gene regulation are only beginning to be unraveled.

The results presented in this paper have important implications for the field of evolutionary developmental biology (evo-devo). A prominent view in this field is that morphological diversity is caused primarily by mutations in the cis-regulatory regions of genes [40], rather than by changes in protein coding sequences as suggested by other authors (eg [41]). A compromising proposal [42] predicts that the relative importance of both cis-regulatory and protein coding changes will vary depending on factors such as the position or rank of a gene in a regulatory network, the population dynamics and the evolutionary time span. In this model, highly interconnected genes are preferentially subjected to cis-regulatory evolution, while mutations in protein coding sequences are more prevalent in genes residing in less densely clustered parts of the network. Our results suggest that intragenic methylation might be an additional constituent of the cis-regulatory machinery regulating the components of densely connected metabolic and information processing networks constitutively expressed in most cells. In contrast, effector genes responsible for cell differentiation and specialization might not require these rich and complex regulatory inputs, and would not be methylated.

To understand the relevance of epigenetic influences on regulatory networks to developmental and evolutionary transitions, studies of the same genes and their interacting partners are required in different phyla. By comparing epigenomes, with their developmental end-points from different phyla we should be able to reveal what is functionally common and what is different. The emerging field of insect epigenomics will undoubtedly accelerate these efforts by providing novel and exciting data on genome-wide analyses of TF-binding sites, histone modifications, DNA methylation and context-dependent gene expression.

## Conclusion

In conclusion, we show that approximately one third of the annotated gene set in *Apis mellifera *is expected to be methylated at the CpG dinucleotides residing in intragenic regions of conserved genes involved in core 'housekeeping' biological functions. Our data suggest that DNA methylation is an ancient epigenetic mechanism that was tailored to be part of modern regulatory networks. Thus, these findings go beyond epigenetics and touch upon the invention of genome-wide regulatory networks in modern animals with important implications for the emergence of organizational complexity during metazoan evolution. We propose to compare epigenomes, with their developmental end-points from different phyla to ascertain what is functionally common and what is different. In this context, the emerging field of insect epigenomics might be particularly useful in unravelling the underlying mechanisms of environmentally-driven phenotypic and behavioural plasticity.

## Methods

### Microarray platform

The details of the first generation *Apis mellifera *genomic array can be found at: . Briefly, long oligos for the array were developed by Debashis Rana and Gos Micklem at Cambridge University , using a modified version of OligoArray 2.1 to identify unique sequences (60-69 mers) each of the bee genes in the honey bee genome project 'official gene set' [43,44] deposited at . A total of 12,915 unique oligos were generated. Details on source sequences, which encompassed honey bee predicted genes, EST's and markers for bee parasites and pathogens available at . Reverse-strand oligos were added for 525 predictions, focusing on EST reads and transcripts predicted for bee pathogens (table two in file ArrayDevelopment.rtf at the above web site). As such, the final set contains 13,439 oligos (sequences in Array_fasta/Oligoset13440.txt at the above web site). The platform contains five types of spots (additional file 3): 13,439 honeybee cDNA probes were printed at least twice, resulting in 26,880 cDNA spots; 25 negative control probes (microbial or plant DNA) printed at least 12 times each (348 negative control spots); 4 positive control probes printed 96 times each (384 positive control spots); 384 spots were left blank and 802 spots were printed with buffer only. The array features are summarized in additional file 3.

### DNA and RNA extractions and other molecular protocols

Bees were collected and dissected as described elsewhere [19]. DNAs were extracted as described in [16] and bisulfite converted using the QIAGEN Epitect Bisulfite Kit [19]. RNA extractions, labelling and array hybridization were performed according to standard protocols with minor modifications [19,33]. With the exception of the RNA cocktail RNA extractions were done in triplicates or in duplicates (HP gland). The cocktail was a mixture of poly-adenylated mRNAs, other preparations were total RNAs. Total RNAs were extracted via the combined Trizol/QIAGEN method [45] and mRNAs purified using the magnetic beads from Dynal. RNA cocktail was a subjective mixture of the following RNAs (the proportion of each RNA in the final pool is shown in brackets): mixed 0-72 hr embryos (1%); mixed larvae, including queen larva (13%); mixed pupae (20%); adult brains, including drone and queen brains (13%); thorax muscles (12%); worker whole abdomens (15%); queen ovaries (15%); testes and queen spermathecae (3%); whole queen (5%); appendages (antennae, legs, wings) (3%); mixed glands (0.1%). Although the cocktail was not taken into account in the ubiquitous/restricted analysis, its hybridization profile served as a useful control to evaluate the 'biological power' of the genomic microarray.

Other molecular protocols including cDNA or cRNA labelling, hybridization, PCR and sequencing are described elsewhere [33,45,46]. Each amplified RNA sample was labelled with Cy3 and independently with Cy5. The labelled samples were mixed and hybridized with individual slides: antennae - 4 replicates (slides); brains - 4 replicates; cocktail - 2 replicates; HPG - 2 replicates; larvae - 4 replicates; ovary - 3 replicates; thorax - 3 replicates. The primers used for amplification of the genes shown in table 1 from bisulfite-treated DNA are shown in additional file 4.

### Microarray analysis

The method chosen for segmenting the images was the fixed circle method (Eisen, ScanAlyze's user manual, 1999, ). This method has been shown to perform with consistent accuracy on both good and bad microarray images [47], but outperforms other methods on images of lower quality. For each spot an intensity value was computed by subtracting the mean foreground intensity to the median background intensity. By detailed inspection of the images we established that the surfaces of blank and buffer spots had different properties than those where DNA probes were printed, probably resulting in different optical behaviours. For this reason, and in order to potentially adjust for cross-hybridization effects, only plant and microbial DNA negative control spots were used to determine an empirical null distribution for each array and channel. Three negative control probes were removed from the analysis as their signal was consistently biased toward high intensities (1-L22585_IVT_6, 1-modified_GFP_39 and 1-Q9LJQ4_IVT_1). A probability of expression, Psca, where 's' denotes the intra-array replicates, 'c' the channel and 'a' the array, was derived for each spot by comparing its intensity to the null distribution (the distribution of the negative controls on the same array, in the same channel). Each gene was printed at least twice on each array, each array had two channels, and at least two hybridisations were conducted for each condition. Thus, the experiment yielded at least eight Psca values for each gene. The median of these Psca values was used as an estimate of the probability of a gene expression for a given experimental condition. Computations were conducted using python and R scripts available from the authors upon request. Since spots are only compared to negative controls on the same array using the same channel, our method allows comparisons of microarrays from different experiments without any normalisation. Other experimental details are shown in additional file 4. ArrayExpress accession: E-MEXP-2093. All scripts used in this work are freely avalibale from the authors.

### CpG analysis

The CpG bias of a sequence is defined as the ratio of the observed frequency of CpG dinucleotides divided by the expected frequency of CpG dinucleotides where the expected number of CpG dinucleotides is the product of the frequency of C and G nucleotides in a given sequence. When no Cs or no Gs are observed, the CpG bias is arbitrarily set to one. CpG islands were identified using Alan Bleasby's (EBI) cpgplot at the Pasteur Institute with default parameters .

## Authors' contributions

RM, RK and SF conceived the study. SF and YP carried out the bioinformatics analysis. RK carried out the microarray experiments. GL conducted the brain microarray experiment. SF and RK participated in discussions and provided valuable suggestions. RM prepared RNA samples and wrote the manuscript. SF and RK helped to draft the manuscript. All authors approved the final manuscript.

## Supplementary Material

Additional file 1 **Proportion of cDNA spots found expressed on each array and for each channel in different experiment**. This PDF displays a graph expressing Proportion of cDNA spots found expressed on each array and for eachchannel in different experiment. Click here for file

Additional file 2 **The correlation between ubiquitous genes and low CpG o/e ratio holds at different thresholds at which genes are considered expressed in microarray experiments**. The columns show three different thresholds for gene presence/absence calls. The first column lists three different questions, the null hypotheses are the "No" answers to these questions. The p-values for the rejection of the null hypotheses are reported in each cell. Click here for file

Additional file 3 **Summary of the types and number of features present on the honey bee oligonucleotide array**. This word document contains a table expressing Summary of the types and number of features present on the honey bee oligonucleotide array. Click here for file

Additional file 4 **Gene ontology**. Supplementary methods: Gene Ontology, Primers for bisulfite sequencing, Array methodology. Click here for file

## References

[B1] van Steensel B (2005). Mapping of genetic and epigenetic regulatory networks using microarrays. Nat Genet.

[B2] Hawkins RD, Ren B (2006). Genome-wide location analysis: insights on transcriptional regulation. Hum Mol Genet.

[B3] Bird A (2002). DNA methylation patterns and epigenetic memory. Genes & Dev.

[B4] Weber M, Schübeler D (2007). Genomic patterns of DNA methylation: targets and function of an epigenetic mark. Curr Opin Cell Biol.

[B5] Jaenisch R, Bird A (2003). Epigenetic regulation of gene expression: how the genome integrates intrinsic and environmental signals. Nat Genet.

[B6] Feil R (2006). Environmental and nutritional effects on the epigenetic regulation of genes. Mutat Res.

[B7] Reik W (2007). Stability and flexibility of epigenetic gene regulation in mammalian development. Nature.

[B8] Rakyan VK, Down TA, Thorne NP, Flicek P, Kulesha E, Gräf S, Tomazou EM, Bäckdahl L (2008). An integrated resource for genome-wide identification and analysis of human tissue-specific differentially methylated regions (tDMRs). Genome Res.

[B9] Zilberman D, Gehring M, Tran RK, Ballinger T, Henikoff S (2007). Genome-wide analysis of Arabidopsis thaliana DNA methylation uncovers an interdependence between methylation and transcription. Nat Genet.

[B10] Tweedie S, Charlton J, Clark V, Bird A (1997). Methylation of genomes and genes at the invertebrate-vertebrate boundary. Mol Cell Biol.

[B11] Regev A, Lamb MJ, Jablonka E (1998). The Role of DNA Methylation in Invertebrates: Developmental Regulationor Genome Defense?. Mol Biol Evol.

[B12] Field LM (2000). Methylation and expression of amplified esterase genes in the aphid Myzus persicae (Sulzer). Biochem J.

[B13] Bestor TH (1990). DNA methylation: evolution of a bacterial immune function into a regulator of gene expression and genome structure in higher eukaryotes. Philos Trans R Soc Lond B Biol Sci.

[B14] Bird AP (1995). Gene number, noise reduction and biological complexity. Trends Genet.

[B15] Goll MG, Bestor TH (2005). Eukaryotic cytosine methyltransferases. Annu Rev Biochem.

[B16] Wang Y, Jorda M, Jones PL, Maleszka R, Robertson HM, Mizzen CA, Peinado M, Robinson GE (2006). Functional CpG Methylation System in a Social Insect. Science.

[B17] Maleszka R (2008). Epigenetic integration of environmental and genomic signals in honey bees: the critical interplay of nutritional, brain and reproductive networks. Epigenetics.

[B18] Schaefer M, Lyko F (2007). DNA methylation with a sting: an active DNA methylation system in the honeybee. Bioessays.

[B19] Kucharski R, Maleszka J, Foret S, Maleszka R (2008). Nutritional control of reproductive status in honey bees via DNA methylation. Science.

[B20] Suzuki MM, Kerr AR, De Sousa D, Bird A (2007). CpG methylation is targeted to transcription units in an invertebrate genome. Genome Res.

[B21] Wang Y, Leung FC (2008). GC Content Increased at CpG Flanking Positions of Fish Genes Compared with Sea Squirt Orthologs as a Mechanism for Reducing Impact of DNA Methylation. PLoS ONE.

[B22] Bork P (2000). Powers and pitfalls in sequence analysis: the 70% hurdle. Genome Res.

[B23] Abbott A (2005). Competition boosts bid to find human genes. Nature.

[B24] Irizarry RA, Warren D, Spencer F, Kim IF, Biswal S, Frank BC, Gabrielson E (2005). Multiple-laboratory comparison of microarray platforms. Nature Methods.

[B25] Peng X, Wood CL, Blalock EM, Chen KC, Landfield PW, Stromberg AJ (2003). Statistical implications of pooling RNA samples for microarray experiments. BMC Bioinformatics.

[B26] Butte AJ, Dzau VJ, Glueck SB (2001). Further defining housekeeping, or "maintenance," genes: Focus on "A compendium of gene expression in normal human tissues". Physiol Genomics.

[B27] Vandesompele J, De Preter K, Pattyn F, Poppe B, Van Roy N, De Paepe A (2002). Accurate normalization of real-time quantitative RT-PCR data by geometric averaging of multiple internal control genes. Genome Biol.

[B28] Gibson G (2008). The environmental contribution to gene expression profiles. Nat Rev Genet.

[B29] Zhu J, He F, Hu S, Yu J (2008). On the nature of human housekeeping genes. Trends Genet.

[B30] Barchuk AR, dos Santos Cristino A, Kucharski R, Simões ZLP, Maleszka R (2007). Molecular determinants of caste differentiation in the highly eusocial honeybee Apis mellifera. BMC Dev Biol.

[B31] Miklos GL, Maleszka R (2001). Integrating molecular medicine with functional proteomics: realities and expectations. Proteomics.

[B32] Miklos GL, Maleszka R (2004). Microarray reality checks in the context of a complex disease. Nat Biotechnol.

[B33] Thompson GJ, Kucharski R, Maleszka R, Oldroyd BP (2008). Genome-wide analysis of genes related to ovary activation in worker honey bees. Insect Mol Biol.

[B34] Wittkopp PJ (2007). Variable gene expression in eukaryotes: a network perspective. J Exp Biol.

[B35] Elango N, Hunt BG, Goodisman MA, Yi SV (2009). DNA methylation is widespread and associated with differential gene expression in castes of the honeybee, Apis mellifera. Proc Natl Acad Sci USA.

[B36] Wang Y, Leung FC (2009). In silico prediction of two classes of honeybee genes with CpG deficiency or CpG enrichment and sorting according to gene ontology classes. J Mol Evol.

[B37] Miklos GL, Maleszka R (2000). Deus ex genomix. Nature Neurosci.

[B38] Lee TI, Young RA (2000). Transcription of eukaryotic protein-coding genes. Annu Rev Genet.

[B39] Di Croce L, Raker VA, Corsaro M, Fazi F, Fanelli M, Faretta M, Fuks F, Lo Coco F, Kouzarides T, Nervi C, Minucci S, Pelicci PG (2002). Methyltransferase recruitment and DNA hypermethylation of target promoters by an oncogenic transcription factor. Science.

[B40] Caroll SB, Grenier JK, Weatherbee SD (2005). From DNA to diversity: molecular genetics and the evolution of animal design.

[B41] Hoekstra HE, Coyne JA (2007). The locus of evolution: evo devo and the genetics of adaptation. Evolution.

[B42] Stern DL, Orgogozo V (2009). Is genetic evolution predictable?. Science.

[B43] The Honeybee Genome Sequencing Consortium (2006). Insights into social insects from the genome of the honeybee *Apis mellifera*. Nature.

[B44] Robinson GE, Evans JD, Maleszka R, Robertson HM, Weaver DB, Worley K, Gibbs RA, Weinstock GM (2006). Sweetness and Light: Illuminating the Honey Bee Genome. Insect Mol Biol.

[B45] Kucharski R, Maleszka R (2005). Microarray and rtPCR analyses of gene expression in the honey bee brain following caffeine treatment. J Mol Neurosci.

[B46] Kucharski R, Maleszka R (2002). Molecular profiling of behavioural development: differential expression of mRNAs for inositol 1,4,5-trisphosphate 3-kinase isoforms in naive and experienced honeybees (*Apis mellifera*). Mol Brain Res.

[B47] Lehmussola A, Ruusuvuori P, Yli-Harja O (2006). Evaluating the performance of microarray segmentation algorithms. Bioinformatics.

[B48] Edwards AWF (2004). Cogwheels of the Mind: the story of Venn diagrams.

[B49] Foret S, Maleszka R (2006). Function and evolution of a gene family encoding odorant binding-like proteins in a social insect, the honey bee (*Apis mellifera*). Genome Res.

