# Supplementary material for: Epigenetic regulation of the honey bee transcriptome: unravelling the nature of methylated genes
Source: BMC Genomics. 2009 Oct 14;10:472. doi: 10.1186/1471-2164-10-472 (PMC2768749; doi:10.1186/1471-2164-10-472)

Additional file 1 - Proportion of cDNA spots found expressed on each array and for each channel in different experiment

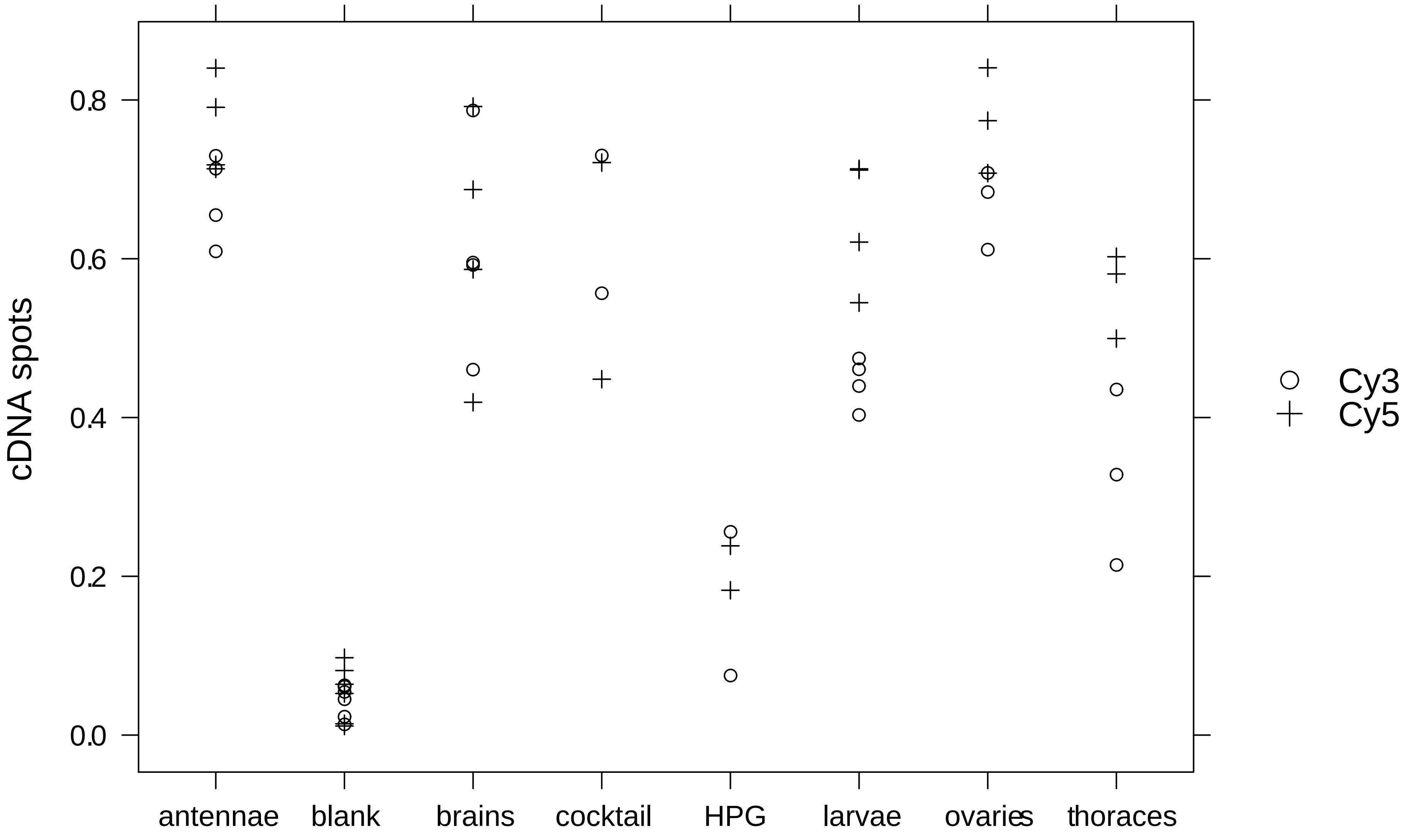

Supplement: Additional file 1 — Proportion of cDNA spots found expressed on each array and for each channel in different experiment. This PDF displays a graph expressing Proportion of cDNA spots found expressed on each array and for eachchannel in different experiment. [file 1471-2164-10-472-S1.PDF]
