# Supplementary material for: Epigenetic regulation of the honey bee transcriptome: unravelling the nature of methylated genes
Source: BMC Genomics. 2009 Oct 14;10:472. doi: 10.1186/1471-2164-10-472 (PMC2768749; doi:10.1186/1471-2164-10-472)
Supplement: Additional file 2 — The correlation between ubiquitous genes and low CpG o/e ratio holds at different thresholds at which genes are considered expressed in microarray experiments. The columns show three different thresholds for gene presence/absence calls. The first column lists three different questions, the null hypotheses are the "No" answers to these questions. The p-values for the rejection of the null hypotheses are reported in each cell. [file 1471-2164-10-472-S2.DOC]

**Additional file 2 - The correlation between ubiquitous genes and low CpG o/e ratio holds at different thresholds at which genes are considered expressed in microarray experiments.**

The columns show three different thresholds for gene presence/absence calls. The first column lists three different questions, the null hypotheses are the “No” answers to these questions. The p-values for the rejection of the null hypotheses are reported in each cell.

|  | 0.1 | 0.05 | 0.01 |
| --- | --- | --- | --- |
| Are ubiquitous and restricted genes associated with different CpG bias (Fisher exact test) | 2.2e-128 | 7.9e-111 | 2.4e-34 |
| Are ubiquitous genes enriched in genes with a CpG bias < 1 (Hypergeometric test) | 3.8e-91 | 3.7e-77 | 1.6e-39 |
| Are restricted genes enriched in genes with a CpG bias > 1 (Hypergeometric test) | 3.7e-63 | 3.0e-51 | 4.4e-3 |
