# Supplementary material for: Epigenetic regulation of the honey bee transcriptome: unravelling the nature of methylated genes
Source: BMC Genomics. 2009 Oct 14;10:472. doi: 10.1186/1471-2164-10-472 (PMC2768749; doi:10.1186/1471-2164-10-472)
Supplement: Additional file 3 — Summary of the types and number of features present on the honey bee oligonucleotide array. This word document contains a table expressing Summary of the types and number of features present on the honey bee oligonucleotide array. [file 1471-2164-10-472-S3.DOC]

**Additional file 3 - Summary of the types and number of features present on the honey bee oligonucleotide array**

| Spot type | Number of different probes | Number of spots per array |
| --- | --- | --- |
| cDNA | 13,439 | 26,880 |
| Positive controls | 4 | 384 |
| Negative controls | 25 | 348 |
| Blank | NA | 384 |
| Buffer | NA | 802 |
