# Supplementary material for: Epigenetic regulation of the honey bee transcriptome: unravelling the nature of methylated genes
Source: BMC Genomics. 2009 Oct 14;10:472. doi: 10.1186/1471-2164-10-472 (PMC2768749; doi:10.1186/1471-2164-10-472)
Supplement: Additional file 4 — Gene ontology. Supplementary methods: Gene Ontology, Primers for bisulfite sequencing, Array methodology. [file 1471-2164-10-472-S4.DOC]

**Additional file 4 - Supplementary methods: Gene Ontology, Primers for bisulfite sequencing, Array methodology**

**GENE ONTOLOGY**

Gene ontology (GO) terms were assigned to honeybee orthologs using Drosophila RefSeq database. For illustration purposes only molecular function level 3 ontology terms (where Level 0 = root = Gene_ontology) were selected and grouped into larger categories as follows:

cell motor protein - microfilament motor activity (GO:0000146), microtubule motor activity (GO:0003777); structural protein - structural constituent of cell wall (GO:0005199), structural constituent of cytoskeleton (GO:0005200), structural constituent of muscle (GO:0008307), structural constituent of peritrophic membrane (GO:0016490), extracellular matrix structural constituent (GO:0005201), structural constituent of cuticle (GO:0042302); ribosomal protein - structural constituent of ribosome (GO:0003735); transcription factor - transcription factor activity (GO:0003700), RNA polymerase II transcription factor activity (GO:0003702), transcription cofactor activity (GO:0003712), transcription initiation factor activity (GO:0016986), transcription activator activity (GO:0016563), transcription repressor activity (GO:0016564); signal transducer - signal transducer activity (GO:0004871); transporter - neurotransmitter transporter activity (GO:0005326), transmembrane transporter activity (GO:0022857); enzyme - peroxidase activity (GO:0004601), transposase activity (GO:0004803), oxidoreductase activity (GO:0016491), transferase activity (GO:0016740), hydrolase activity (GO:0016787), lyase activity (GO:0016829), isomerase activity (GO:0016853), ligase activity (GO:0016874), electron transporter, transferring electrons within CoQH2-cytochrome c reductase complex activity (GO:0045153); protein binding - protein binding (GO:0005515); nucleic acid & chromatin binding - nucleic acid binding (GO:0003676), chromatin binding (GO:0003682); other binding - cofactor binding (GO:0048037), odorant binding (GO:0005549), lipid binding (GO:0008289), carbohydrate binding (GO:0030246), nucleotide binding (GO:0000166).

**PRIMERS FOR BISULFITE SEQUENCING**

| **Gene fragment** | **PCR round** | **Primer ID** | **Sequence** |
| --- | --- | --- | --- |
| dynactin | first | 4.5-F1 | GGATTAATATTTTAATTGATT |
|  |  | 4.5-R1 | ATATAAACTTACAATACAAC |
|  | nested | 4.5-F3 | GCAGAATTCAGAAAAATAGTAATGTGAGA |
|  |  | 4.5-R3 | CGCAAGCTTCAAAATATATACAAAAATCTAAT |
| MTM (exon 5) | first | MTM-F1 | AAGTAGTTAATTAATGTGTGGTAT |
|  |  | MTM-R1bis | CTTCTTAATAAAAACACTAAACAA |
|  | nested | MTM-F2 | GCAGAATTCTGTATAAGTTTAAAGTTTTAGAGT |
|  |  | MTM-R2bis | CGCAAGCTTTTATCAAATCTTAAAATCCACTAA |
| MTM (exon 7) | first | MTMex7-F1 | TTAGTGTTGTATTTATTAGTATAT |
|  |  | MTMex7-R1 | TACAATATCTACRATCTACATCAA |
|  | nested | MTMex7-F2 | GCAGAATTCGTGGAAAGAATTTTATTTTGGTTA |
|  |  | MTMex7-R2 | CGCAAGCTTTCTAAACTTAATTATACTATACAA |
| HMT | first | HMT-F1bis | AATATATTGAGAATGGTAAGAATT |
|  |  | HMT-R1bis | CCATAATATTCTCATCAATAAAAT |
|  | nested | HMT-F2bis | GCAGAATTCATTGAAGAAGATATAGATGGTGTT |
|  |  | HMT-R2bis | CGCAAGCTTTAAATTCTCCTAATTTAACACAAA |
| nadrin | first | nadrin2-F1 | GTAGTTATTGTTAGTGGATTTAAT |
|  |  | nadrin2-R1 | AATATAATAATCACTTTTTTACAA |
|  | nested | nadrin2-F2 | GCAGAATTCTATTTAGTGGATAAATAATAGGTT |
|  |  | nadrin2-R2 | CGCAAGCTTATCCTAAAAATTAATACTTTCATA |
| PKCbp1 | first | PKCbp1-F1 | TATTATGGTGTTTAAGATTTAGTT |
|  |  | PKCbp1-R1 | TTCTCATATACATTAACCAATATA |
|  | nested | PKCbp1-F2 | GCAGAATTCGGAAGAGGTATATAAGGAAGTAGT |
|  |  | PKCbp1-R2bis | CGCAAGCTTTCTACCACACATTACACACCATTA |
| TBP | first | TBP-F1 | ATGTATTAGTTGTGAGTATTGTTT |
|  |  | TBP-R1 | CATATATCACTTACTATAAACAAA |
|  | nested | TBP-F2 | GCAGAATTCTAAATAAGATGATTGTAATTGTAT |
|  |  | TBP-R2 | CGCAAGCTTCACTTCTTTTATACTATTAACTAA |
| CKIIb | first | CKIIb-F1 | AGATATGATTTTTGATTTTGAATT |
|  |  | CKIIb-R1 | TTCAACTTACCTAAATACAAACTA |
|  | nested | CKIIb-F2 | GCAGAATTCGATGAATTGGATGGTTATTTAAAT |
|  |  | CKIIb-R2 | CGCAAGCTTTACTTCTCCAAAAACATCACTAAA |
| NTE | first | NTE-F2 | GCAGAATTCAGGTAGTATTATAATGGAATTTGT |
|  |  | NTE-R2 | CGCAAGCTTAACAAATCTACTAAAATCACTATA |
|  | nested | NTE-F2bis | GCAGAATTCGGTTTGTATAATAAGAAGATTAAT |
|  |  | NTE-R2bis | CGCAAGCTTTCTTACAATTCTATACTAAAATCT |
| GLOX (5'end) | first | GOX5_sFOR_1 | GATTAAATATATYGATTGGGATTA |
|  |  | GOX5_sREV_1 | TTCRAAATACCATATCRAATTACR |
|  | nested | GOX5_sFOR_2 | GCAGAATTCGTTTTYGAGATTTTATTAGATTYG |
|  |  | GOX5_sREV_2 | CGCAAGCTTCCAACRATTAATAATAAACAACRC |
| GLOX (3'end) | first | GOX3_sFOR_1 | GTATTAYGTTGAATAGTAAAGATT |
|  |  | GOX3_sREV_1 | CTCRCCRACCATATTAACAAAAAC |
|  | nested | GOX3_sFOR_2 | GCAGAATTCTTATTTGGAGTAAYGATTTGGTTA |
|  |  | GOX3_sREV_2 | CGCAAGCTTAAATTACCCRAAATCACCTAATAA |
| VHDL | first | EPI01_sFOR_1 | AAGAATTAAATGAGTTATGATAGT |
|  |  | EPI01_sREV_1 | TATATATAATCACCAAACATCAAA |
|  | nested | EPI01_sFOR_2 | GCAGAATTCGATAGTATGTATATTGAATGTTAT |
|  |  | EPI01_sREV_2 | CGCAAGCTTATAACACCATTCCAATTAATAATA |
| OBP13 | first | EPI02_sFOR_1 | TTTTTAGGTAGTTTTTGAAGAATT |
|  |  | EPI02_sREV_1 | ATCTTCATCAAATTATTATCTATA |
|  | nested | EPI02_sFOR_2 | GCAGAATTCGAAGAAAATGGTATTGATTTAAGT |
|  |  | EPI02_sREV_2 | CGCAAGCTTTAAAACATTACAATAATTTACTAA |
| RIKEN DB777978 | first | EPI03_sFOR_1 | TTGAAATTTAGAATGAAATTTGTT |
|  |  | EPI03_sREV_1 | AATACACAAATTTATTCCAAATTA |
|  | nested | EPI03_sFOR_2 | GCAGAATTCGTGAGATTTTTGTTTGATTTATTT |
|  |  | EPI03_sREV_2 | CGCAAGCTTAATTTATTCCAAATTATTACTTAA |
| ImpL3-like | first | EPI06_sFOR_1 | TAATGATTTGTTGTGATAATGTGT |
|  |  | EPI06_sREV_1 | TAAACAAACAATAAACAAATTCAA |
|  | nested | EPI06_sFOR_2 | GCAGAATTCGTTATGAGTGAAAGTTGTGTGTTT |
|  |  | EPI06_sREV_2 | CGCAAGCTTCACATAAAACATACACAAAACACA |
| squid | first | EPI07A_sFOR_1 | GGTGATATTGAAAGTATTAATGTT |
|  |  | EPI07A_sREV_1 | CCAAATACACTAAAACAATAACAA |
|  | nested | EPI07A_sFOR_2 | GCAGAATTCTTTAGATTATGGTAGTTGGTGATT |
|  |  | EPI07A_sREV_2 | CGCAAGCTTATCCTACACAACAATAAACACATA |

**Array technology**

ArrayExpress accession: E-MEXP-2093

HYBRIDIZATION

Probe composition: typically 5 ug cRNA (combined) and 40 pmoles of each coupled Cy3 and Cy5, 5xSSC, 0.1% SDS and 25% formamide in a volume of 40 ul. Hybridizations were carried out in CMT Hybridization Chambers (Corning, cat.#2551) in water bath at 42 deg C. for 16 hours. Slides were washed 2x in 2xSSC, 0.1 SDS, 2x in 2xSSC and 2x in 0.1xSSC and dried by spinning in a centrifuge.

LABELING

4 ug aRNA samples in 9 ul of 0.05M carbonate buffer, pH 9.0, were mixed with 2 ul of fresh dye solution prepared by dissolving a vial of mono-reactive cyanine dye (GE Healthcare Life Sciences, Cy3 cat.#PA23001, Cy5 cat.#PA25001) in 20 ul of absolute DMSO (Sigma, cat.#41647) and incubated in darkness at room temperature for 1 hour. Reactions were quenched by adding 4.5 ul 4M hydroxylamine (Sigma, cat.# 159417) and incubating for 15 minuter at RT. Pairs of probes to be used for each hybridization were then combined and purified with QIAGEN's RNeasy MinElute Clanup Kit. Probe quality was analysed using Nanodrop (microarray mode).

SAMPLE COLLECTION/GROWTH

Larvae and pupae were isolated from brood comb cells and sorted according to their developmental stage (number of moults for larvae, and eye and body colour for pupae). To obtain worker honeybees of a known age a single brood frame was removed from a hive and incubated in darkness at 31 C (80% humidity). Newly emerged insects were labelled with a dot of paint and returned to the hive. Young, non-foraging bees were collected at desired time by removing labelled insects from the hive. Foraging honeybee workers were captured near the hive entrance. To ensure that fully matured workers were harvested, only those that carried pollen or nectar were selected. We estimate their age to vary from 20-35 days. All specimens were snap-frozen in liquid nitrogen and stored at -80 degrees C before dissecting body parts and tissues for RNA extractions.

To obtain worker honeybees of known age a single frame of capped brood was removed from a hive and incubated in darkness at 31 C (80% humidity). Emerged adult insects were collected using a pair of tweezers and transferred to a cage containing a tube of fresh honey. Each cage contained up to 75 bees. Bees were killed by rapid freezing in liquid nitrogen and stored at -80 degrees C before dissecting body parts and tissues for RNA extractions.
(Parameters: time unit = seconds, temperature unit = C)

1st instar larvae were removed from the comb and transferred to 48-well microtitre plates with 200ul of larval food per well and kept in a plastic desiccator (BelArt) over saturated solution of potassium sulphate. The temperature was maintained at 34.5 deg C and the humidity was >80%. Larvae were fed twice a day (morning and afternoon) with 50-80ul of larval food per well. Larval food used was a modified Rembold and Lackner basic diet consisting of a mixture (w/w) of 2 parts Royal Jelly and 1 part of the following solution: 3% Yeast extract, 18% Fructose, 18% Glucose.

NUCLEIC ACID EXTRACTION

mRNA was purified from total RNA (see http://www.microarray.adelaide.edu.au/protocols/rna/ for the Trizol/QIAGEN RNeasy total RNA extraction protocol) using Dynabeads following manufacturerï¾’s protocol. mRNA (up to 2 ug per sample) was converted (amplified) to complementary RNA using Agilent's Low Input RNA Fluorescent Linear Amplification Kit (cat.#5184-3523). 1st step - cDNA synthesis from total RNA - was performed as per manufacturer's specifications. 2nd step - aRNA synthesis - was modified in order to incorporate aminoallyl nucleotides: the ribonucleotide mix was replaced with a mixture containing 8 ul of 20 mM each rATP, rCTP and rGTP (Promega), 3 ul of 20 mM rUTP (Promega) and 5 ul of 20 mM aminoallyl UTP (Sigma cat.# A5660). Final rNTP concentration was 2 mM, aaUTP/UTP ratio 5:3. aRNA was purified with QIAGEN's RNeasy MinElute Cleanup Kit (cat.# 74204).

ARRAY MANUFACTURING

UIUC Honey bee oligo 13K v1 Array Protocol May 13, 2007 Array Development text by Jay D. Evans, Gene E. Robinson and Gos Micklem. This document describes the features on a first-generation oligonucleotide microarray developed for the honey bee genome. Funding for this project was provided by USDA-National Research Initiative grant AG2004-36504-14277 (G.E. Robinson, PI, M. Band, J.D. Evans, G. deGrandi Hoffman, K.P. White, Co-PIs) “Honey Bee Applied Genomics and Development of a Whole-Genome Array”. The developmental files can be accessed at http://www.biotech.uiuc.edu/centers/Keck/Functional_genomics/Honey%20Bee%20Oligo.htm Input sequences: A total of 13,145 sequences were used to design oligos, as detailed below: 1) A set primarily from the Honey Bee Genome Sequencing Consortium ‘Official Gene Set’ (circa 11/2005) (N = 10620). 2) Variable exons from the antimicrobial peptide apidaecin (Genbank and Evans, J.D., unpublished) (N = 11). 3) Variable exons from the IG-family gene Dscam (N = 81). 4) miRNA precursor candidates from the bee genome (Weaver, D.B., et al., submitted) (N = 81). 5) non-OGS EST’s from a subtractive library biased toward larval genes upregulated with exposure to the bacterial pathogen Paenibacillus larvae, Evans, J.D., unpublished. RNA was derived from 1st-instar honey bee larvae challenged with bacteria as described in Evans and Pettis, 2005, Evolution, 59(10), 2270-2274 (N=81). 6) Non-OGS EST’s from the Univ. Illinois bee brain EST project (Whitfield, C. W., Band, M. R., Bonaldo, M. F., Kumar, C. G., Liu, L., Pardinas, J. R., Robertson, H. M., Bento Soares, M. & Robinson, G. E., 2002, Genome Research, 12, 555-566.) (N=2271). 7) Representative genes from viral, fungal, bacterial, and microsporidian pathogens of honey bees (all in Genbank, ID’s in fasta file) (N=22). Oligo Design: Long oligos for the array were developed by Debashis Rana and Gos Micklem (http://www.gen.cam.ac.uk/Research/micklem.htm) at Cambridge University, using a modified version of OligoArray 2.1 in an iterative process to identify unique sequences (60-69mers) from each of the described (above) bee-related genes and gene fragments. The set of oligos was selected to have as tight a melting temperature distribution as possible, and to avoid repetitive sequences and other anomalies. A total of 12,915 unique oligos were generated (see below for redundancies) representing all but three of the 13,145 source sequences. Of those three (the pathogen gene PlDNAk, the EST sequence JDEA05_1Def3, and the candidate miRNA precursor HCmir13a), the EST and miRNA were represented by 98% identical oligos in the array. Reverse strand oligos were added for 525 predictions, focusing on EST reads and transcripts predicted for bee pathogens (EST – 415; miRNA – 57; OGS – 31, and pathogen – 22). As such the final set contains 13,440 oligos (sequences in Array_fasta/Oligoset13440.txt). The design process was similar to that of the INDAC long oligo set designed for the fruit fly Drosophila melanogaster and available at: http://www.flymine.org/release-5.0/aspect.do?name=INDAC and http://www.flychip.org.uk/services/core/FL002. Oligo and Sequence Redundancy: Distinctly numbered oligos had the same or similar sequences 69 times (>59/69 nt alignment, < 2 mismatches). Different source sequences matched identical oligos (>59/69, < 2 mismatch) 100 times, 44 of which were not genes with predicted splice variants (which were redundant in OGS). 18 were gene calls with splice variants for which oligos matched each variant. 639 source sequences showed matches at the sequence level but did not have identical oligo matches. Of these 524 reflect either splice variants or shared exons (e.g., Dscam exons vs. an entire proposed transcript). 115 are not indicated as splice variants and these appear to be redundant sequences in the source files, either from multiple predictions in OGS or from unrecognized similarity between EST’s and other EST’s or OGS. Most redundancies were single pairs, although one oligo sequence was similar across 6 distinctly called oligos. Printing the Array text by Mark Band and Al Bari Oligos were synthesized by Invitrogen (San Diego, CA) and aliquoted into 384 well plates in Sodium Phosphate buffer. Final concentration of the oligos was 20 uM (micromolar). Arrays were printed on Corning UltraGAPS slides using an Omingrid 100 printing robot (Genomic Solutions, Ann Arbor Michigan) with Arrayit SMP2.5 capillary pins. Following printing slides were stored in vacuum bags purged with Argon gas. Creating the Array Design File (ADF) text by Amro Zayed To ensure that the ADF contains the latest annotation, we first blasted all Honey bee oligos against: 1) The prerelease updated version of OGS v2 (http://racerx00.tamu.edu/downloadFASTA.html - circa 4/9/2007), 2) NCBI’s gene predictions (Ref RNA) for the Honey bee (circa 4/18/2007), 3) All honey bee derived EST sequences on NCBI (circa 4/25/2007), and 4) The latest assembly of the Honey bee genome (AMEL 4.0, circa 4/25/2007). We used blastn with no filtering and we initially retained all matches with an evalue smaller than 1e-3. We then removed blast hits that had an alignment length that is > 4 bp less then the oligo length and/or had an alignment identity less than 95%. Except for the control groups and the Pathogen set, we assigned an oligo’s “Reporter Name”, regardless of which set it was originally designed from, based on the best blast match to the above mentioned databases, assigning priority in the following order: prerelease OGS v2, NCBI’s gene predictions, EST sequence names, and AMEL 4.0 assembly location. In cases where an oligo matched more than one sequence in the prerelease OGS v2 or NCBI’s gene predictions at the same evalue, we assigned the “Reporter Name” and corresponding database accession ID to the gene with the highest numerical value, but included the list of equally matching genes in the “Reporter Comment” field. If an oligo matched to both prerelease OGS v2 and NCBI’s gene prediction, we assigned the “Reporter Name” as the OGS v2 gene name followed by the definition line from NCBI’s gene prediction, in addition to assigning both accession numbers to the oligo. When an oligo matched a prerelease OGS v2 gene, we added its Drosophila ortholog as computed by C. Elsik (http://racerx00.tamu.edu/bee_resources.html) in the “Reporter Comment”. We also used blastp to query the Honey bee gene against Drosophila melanogaster v.5.1 genes (http://flybase.bio.indiana.edu/ circa 5/1/2007), and the best match was also reported in the “Reporter Comment”. For oligos that did not match entries in any of the above mentioned databases, we retained the original annotation information used to design the oligo. Similarly, we retained all the annotation information from the design files for the control sequences and for the Pathogen set.

NUCLEIC ACIDS EXTRACTION/PREPARATION

Total RNA was purified using typical Trizol/QIAGEN RNeasy procedure
(http://www.microarray.adelaide.edu.au/protocols/rna/).

RNA (5 ug per sample) was converted (amplified) to complementary RNA using Agilent's Low Input RNA Fluorescent Linear Amplification Kit (cat.#5184-3523). 1st step - cDNA synthesis from total RNA - was performed as per manufacturer's specifications. 2nd step - aRNA synthesis - was modified in order to incorporate aminoallyl nucleotides: the ribonucleotide mix was replaced with a mixture containing 8 ul of 20 mM each rATP, rCTP and rGTP (Promega), 3 ul of 20 mM rUTP (Promega) and 5 ul of 20 mM aminoallyl UTP (Sigma cat.# A5660). Final rNTP concentration was 2 mM, aaUTP/UTP ratio 5:3.
aRNA was purified with QIAGEN's RNeasy MinElute Cleanup Kit (cat.# 74204).

RNA POOL

The standard ï¾“Cocktail mRNAï¾” is a mixture of mRNA preps extracted from: mixed 0-72hrs embryos (1%); mixed larvae, including queen larva (13%); mixed pupae (20%); adult brains, including drone and queen brains (13%); thorax muscles (12%); worker whole abdomens (15%); queen ovaries (15%); testis and queen spermatheca (3%); whole queen (5%); appendages (antennae, legs, wings) (3%); mixed glands (0.1%).

SCANNING AND IMAGE ACQUISITION

Slides were scanned at 10 um resolution, 600V PMT voltage, 80% laser power.

The method chosen for segmenting the images was the fixed circle method (Michael Eisen, 1999, ScanAlyze user manual, http://rana.lbl.gov/eisen).

The following are the data file header descriptions.

ID: unique spot identifier

MetaRow: row coordinate of the grid (aka subarray)

MetaColumn: column coordinate of the grid (aka subarray)

Row: row coordinate of the spot within the grid

Column: column coordinate of the spot within the grid

spot_radius: radius of the circle delimiting the foreground region

nb_pixels_fg: number of pixels in the foreground region

nb_pixels_bg: number of pixels in the background region

quality_1: A for accepted, R for rejected (Cy3)

fg_mean_1: mean foreground intensity (Cy3)

bg_mean_1: mean background intensity (Cy3)

fg_median_1: median foreground intensity (Cy3)

bg_median_1: median background intensity (Cy3)

fg_stddev_1: foreground intensity standard deviation (Cy3)

bg_stddev_1: background intensity standard deviation (Cy3)

fb_windz_mean_1: windzorised mean foreground intensity (Cy3)

bg_windz_mean_1: windzorised mean background intensity (Cy3)

fg_windz_stddev_1: windzorised foreground intensity standard deviation (Cy3)

bg_windz_stddev_1: windzorised background intensity standard deviation (Cy3)

MW_stat_1: Mann-Whitney statistics of foreground vs background (Cy3)

KS_stat_1: Kolmogorov-Smirnov statistics of foreground vs background (Cy3)

KS_prob_1: p-value of the KS test (Cy3)

quality_2: A for accepted, R for rejected (Cy5)

fg_mean_2: mean foreground intensity (Cy5)

bg_mean_2: mean background intensity (Cy5)

fg_median_2: median foreground intensity (Cy5)

bg_median_2: median background intensity (Cy5)

fg_stddev_2: foreground intensity standard deviation (Cy5)

bg_stddev_2: background intensity standard deviation (Cy5)

fb_windz_mean_2: windzorised mean foreground intensity (Cy5)

bg_windz_mean_2: windzorised mean background intensity (Cy5)

fg_windz_stddev_2: windzorised foreground intensity standard deviation (Cy5)

bg_windz_stddev_2: windzorised background intensity standard deviation (Cy5)

MW_stat_2: Mann-Whitney statistics of foreground vs background (Cy5)

KS_stat_2: Kolmogorov-Smirnov statistics of foreground vs background (Cy5)

KS_prob_2: p-value of the KS test (Cy5)


A probability of expression, Psca, where ï¾‘sï¾’ denotes the intra-array replicates, ï¾‘cï¾’ the channel and ï¾‘aï¾’ the array, was derived for each spot by comparing its intensity to the null distribution (the distribution of the negative controls on the same array, in the same channel). The median of these Psca values was used as an estimate of the probability of a gene expression for a given experimental condition. A gene was called ï¾‘expressedï¾’ in an experimental condition if its median Psca was larger than 95%.
(Parameters: Scanning hardware = GenePix Professional 4200A [Axon Instruments], Scanning software = GenePix Pro [Axon Instruments])
